# The genome sequence of the Mournful Wasp, *Pemphredon lugubris *(Fabricius, 1793)

**DOI:** 10.12688/wellcomeopenres.20948.1

**Published:** 2024-02-23

**Authors:** Steven Falk, Liam M. Crowley, University of Oxford and Wytham Woods Genome Acquisition Lab, Darwin Tree of Life Barcoding collective, Wellcome Sanger Institute Tree of Life Management, Samples and Laboratory team, Wellcome Sanger Institute Scientific Operations: Sequencing Operations, Wellcome Sanger Institute Tree of Life Core Informatics team, Tree of Life Core Informatics collective, Darwin Tree of Life Consortium

**Affiliations:** 1 Independent researcher, Kenilworth, England, UK; 2 University of Oxford, Oxford, England, UK

**Keywords:** Pemphredon lugubris, Mournful Wasp, genome sequence, chromosomal, Hymenoptera

## Abstract

We present a genome assembly from an individual male *Pemphredon lugubris* (the Mournful Wasp; Arthropoda; Insecta; Hymenoptera; Crabronidae). The genome sequence is 328.1 megabases in span. Most of the assembly is scaffolded into 5 chromosomal pseudomolecules. The mitochondrial genome has also been assembled and is 15.88 kilobases in length. Gene annotation of this assembly on Ensembl identified 10,335 protein coding genes.

## Species taxonomy

Eukaryota; Metazoa; Eumetazoa; Bilateria; Protostomia; Ecdysozoa; Panarthropoda; Arthropoda; Mandibulata; Pancrustacea; Hexapoda; Insecta; Dicondylia; Pterygota; Neoptera; Endopterygota; Hymenoptera; Apocrita; Aculeata; Apoidea; Crabronidae; Pemphredoninae; Pemphredonini; Pemphredonina; *Pemphredon; Pemphredon lugubris* (Fabricius, 1793) (NCBI:txid2495172).

## Background

The Mournful Wasp, *Pemphredon lugubris*, is a dark, medium-sized (7.5–12 mm) solitary wasp in the family Pemphredonidae. It occurs across Europe and North America and is common and widespread in the UK. It is the largest and the most frequently recorded *Pemphredon* species in the UK and the only one from the subgenus *Pemphredon* present. Other subgenera of *Pemphredon* are currently in a state of taxonomic flux, although *Pemphredon Pemphredon* remains relatively stable. The integument is entirely black in colour, from which the common name is derived, and it has a petiolate abdomen. The forewing has two submarginal cells, with vein 2m-cu meeting the second submarginal, differentiating it from all other *Pemphredon* species in the UK except *P. morio*. It can be distinguished from this species by the lack of a tooth beneath the base of the antennae and a non-emarginate clypeus.

It occurs across a variety of habitats and may be found anywhere there is suitable deadwood. Nests are constructed in cavities in dead and decaying wood and are typically formed of a main tunnel with several subsidiary tunnels branching off, each with up to several cells ( Bitsch *et al.*, 2001). Females hunt aphids, provisioning each cell with up to 40 ( Lomholdt, 1975). Other Hemipteran prey may be occasionally used ( Whitehead, 1990), but this remains uncertain due to the high prevalence of nest usurpation by *Pemphredon*. It is a univoltine species with a flight period from May to September. It is attacked by the kleptoparasites *Pseudomalus violaceus* and *auratus* ( Blösch, 2000), which gain access to the *Pemphredon* nests by laying eggs on aphids which are subsequently taken by Pemphredon ( Bitsch, 2022).

The complete genome sequence for this species will facilitate studies into the evolution of hunting strategies, sociality, reproductive systems and Hymenopteran taxonomy.

## Genome sequence report

**Figure 1.**
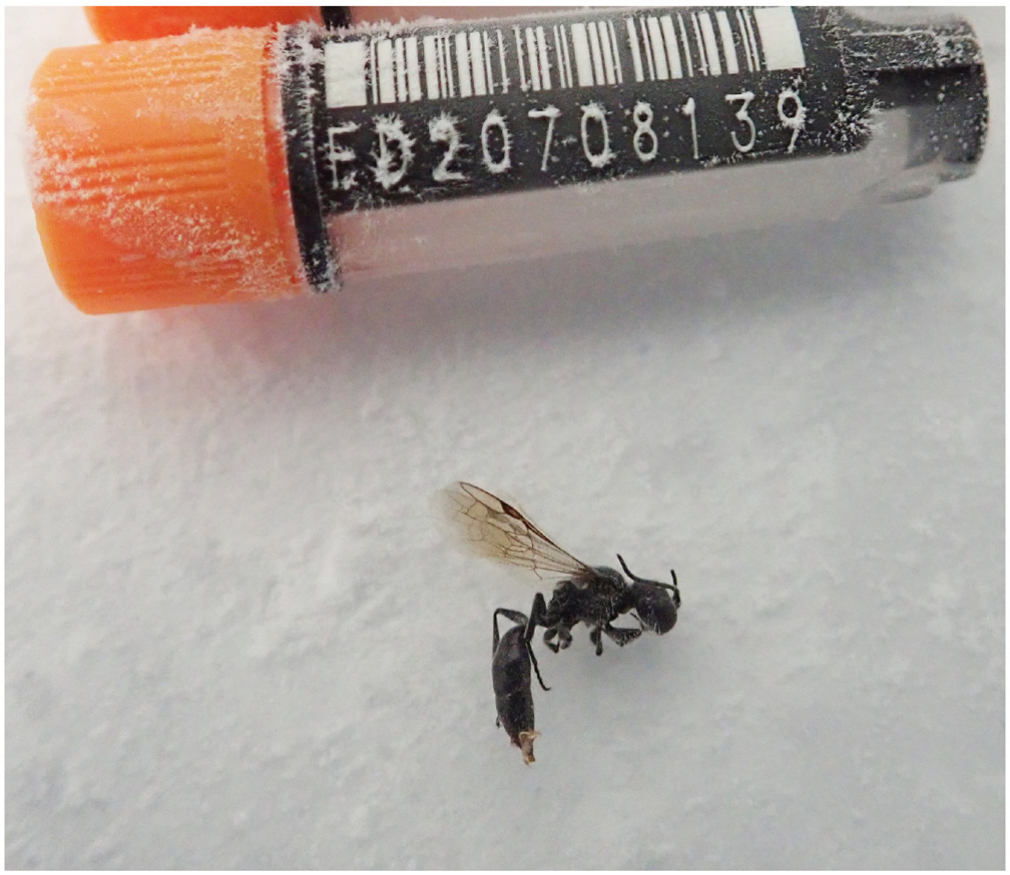
Photograph of the *Pemphredon lugubris* (iyPemLugu1) specimen used for genome sequencing.

The genome was sequenced from one male *Pemphredon lugubris* ( Figure 1) collected from Wytham Woods, Oxfordshire (biological vice-county Berkshire), UK (51.77, –1.31). A total of 58-fold coverage in Pacific Biosciences single-molecule HiFi long reads and 82-fold coverage in 10X Genomics read clouds was generated. Primary assembly contigs were scaffolded with chromosome conformation Hi-C data. Manual assembly curation corrected 421 missing joins or mis-joins, reducing the assembly length by 0.03% and the scaffold number by 23.58%, and increasing the scaffold N50 by 118.68%.

**Figure 2.**
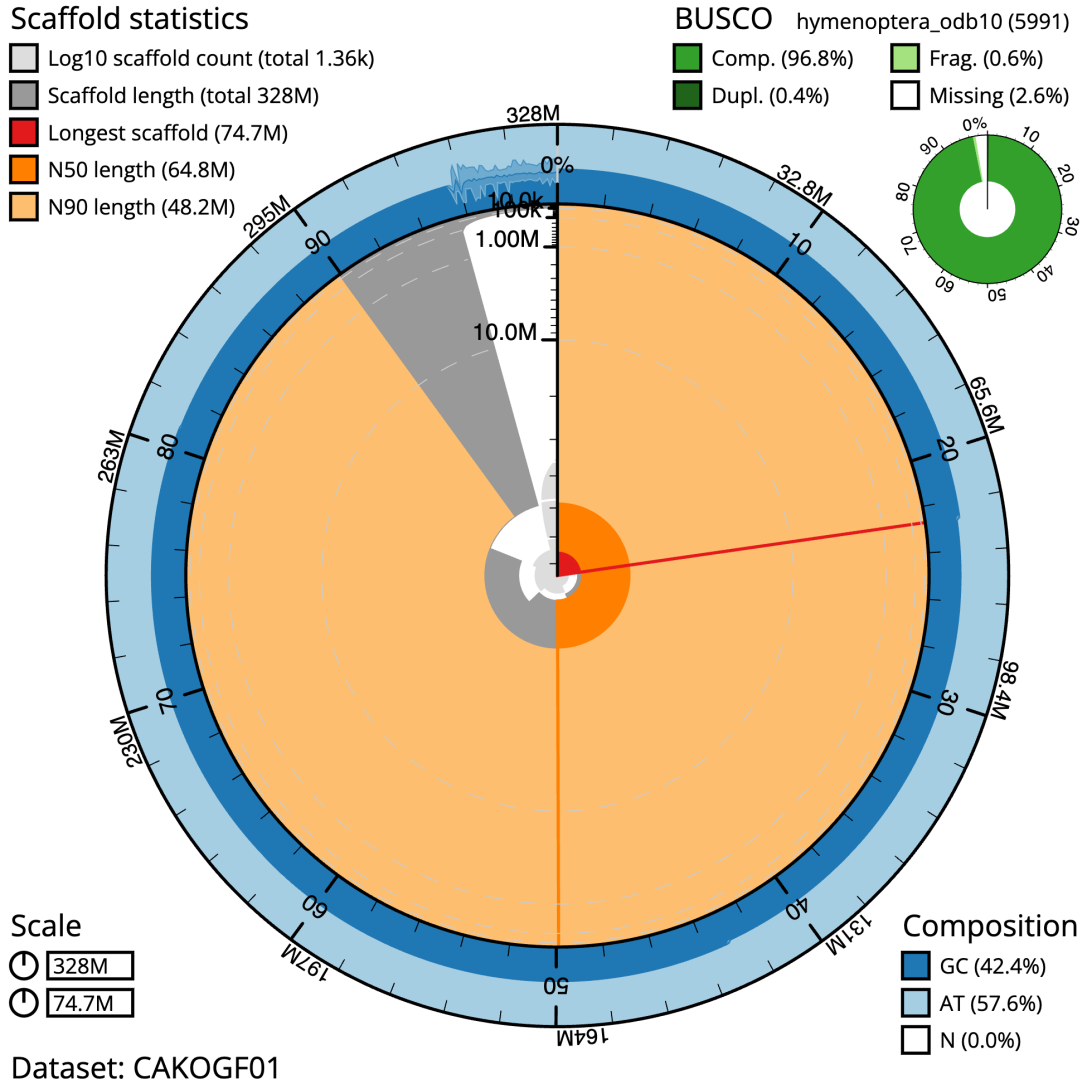
Genome assembly of *Pemphredon lugubris*, iyPemLugu1.1: metrics. The BlobToolKit Snailplot shows N50 metrics and BUSCO gene completeness. The main plot is divided into 1,000 size-ordered bins around the circumference with each bin representing 0.1% of the 328,159,117 bp assembly. The distribution of scaffold lengths is shown in dark grey with the plot radius scaled to the longest scaffold present in the assembly (74,671,469 bp, shown in red). Orange and pale-orange arcs show the N50 and N90 scaffold lengths (64,765,430 and 48,191,648 bp), respectively. The pale grey spiral shows the cumulative scaffold count on a log scale with white scale lines showing successive orders of magnitude. The blue and pale-blue area around the outside of the plot shows the distribution of GC, AT and N percentages in the same bins as the inner plot. A summary of complete, fragmented, duplicated and missing BUSCO genes in the hymenoptera_odb10 set is shown in the top right. An interactive version of this figure is available at https://blobtoolkit.genomehubs.org/view/CAKOGF01/dataset/CAKOGF01/snail.

**Figure 3.**
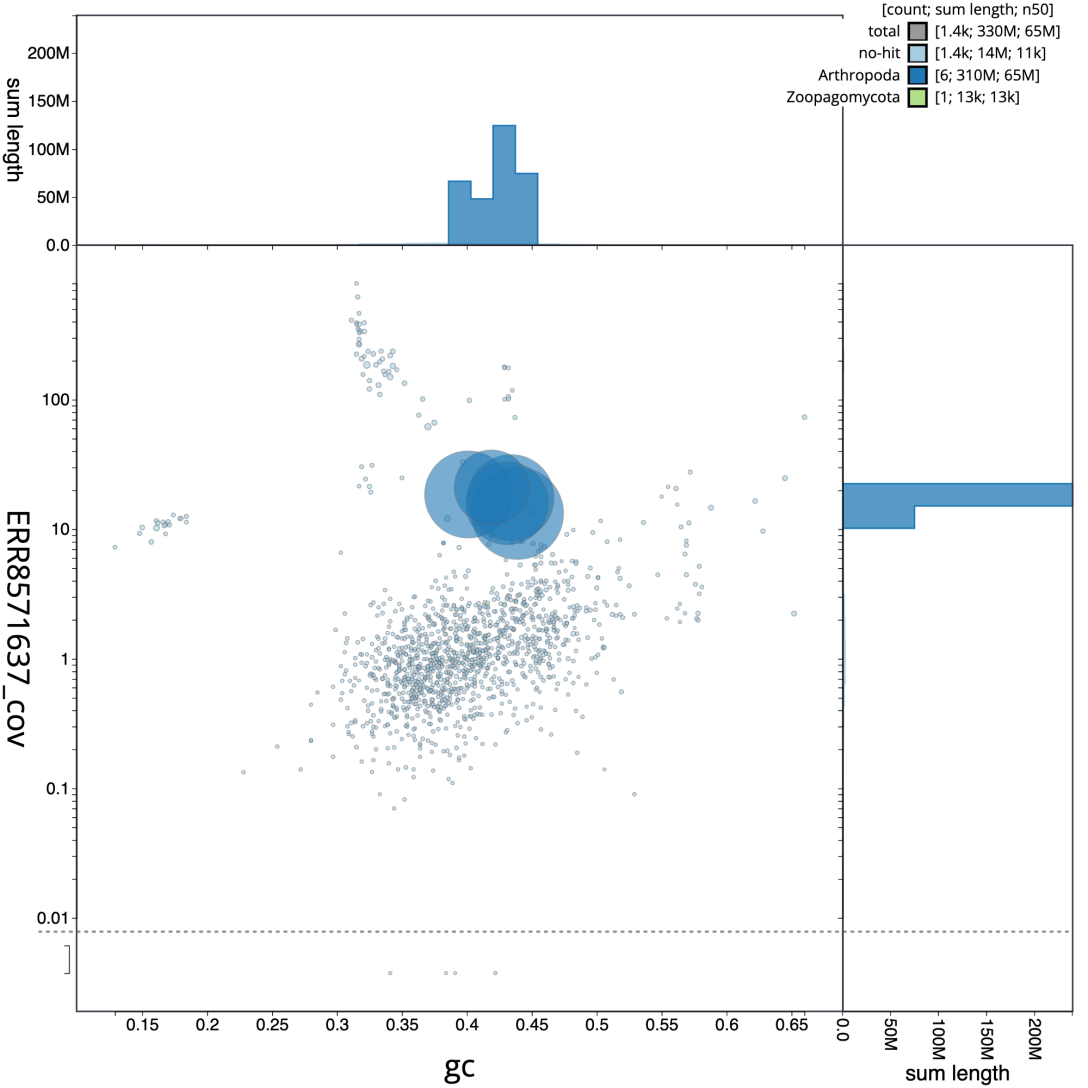
Genome assembly of *Pemphredon lugubris*, iyPemLugu1.1: BlobToolKit GC-coverage plot. Scaffolds are coloured by phylum. Circles are sized in proportion to scaffold length. Histograms show the distribution of scaffold length sum along each axis. An interactive version of this figure is available at https://blobtoolkit.genomehubs.org/view/CAKOGF01/dataset/CAKOGF01/blob.

**Figure 4.**
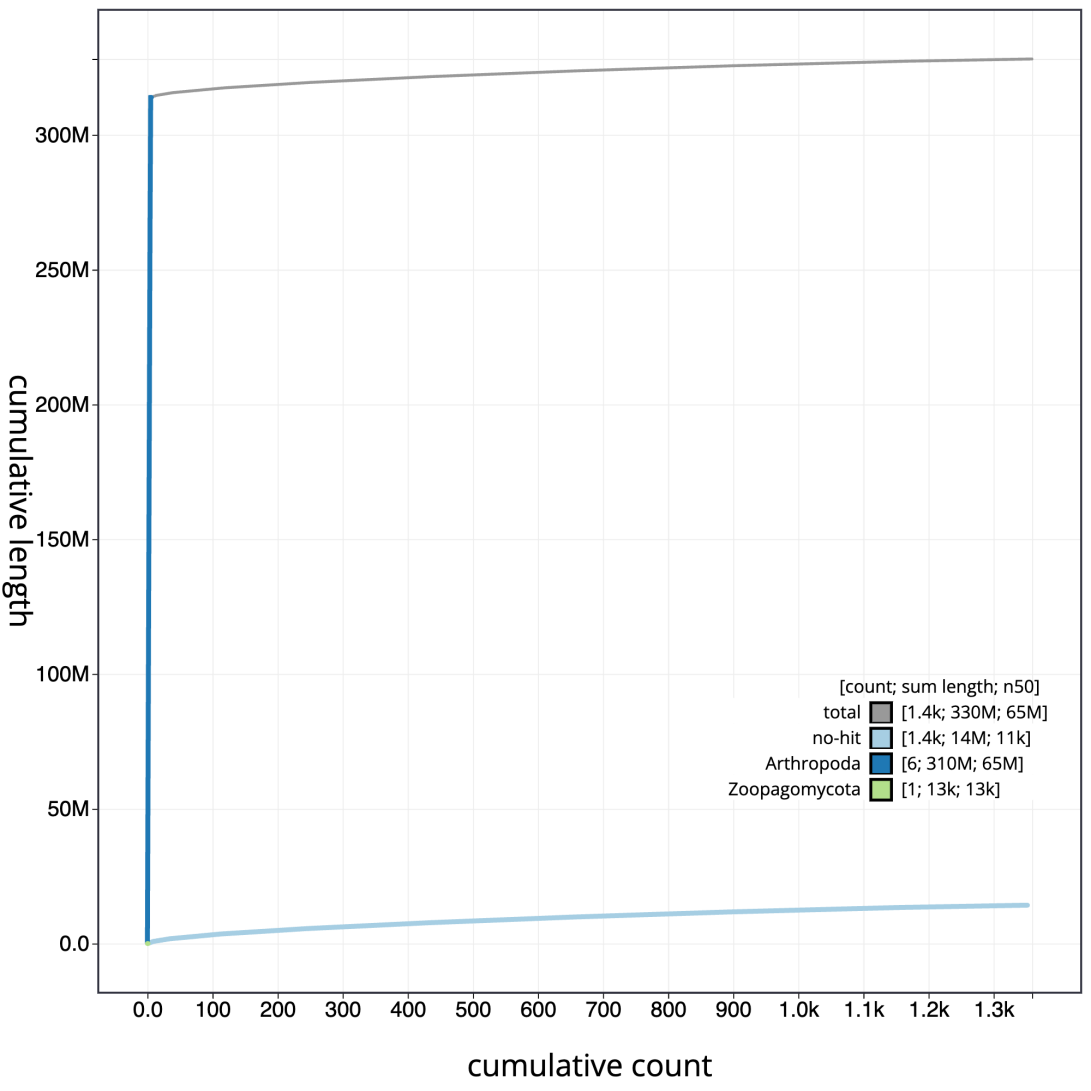
Genome assembly of *Pemphredon lugubris*, iyPemLugu1.1: BlobToolKit cumulative sequence plot. The grey line shows cumulative length for all scaffolds. Coloured lines show cumulative lengths of scaffolds assigned to each phylum using the buscogenes taxrule. An interactive version of this figure is available at https://blobtoolkit.genomehubs.org/view/CAKOGF01/dataset/CAKOGF01/cumulative.

**Figure 5.**
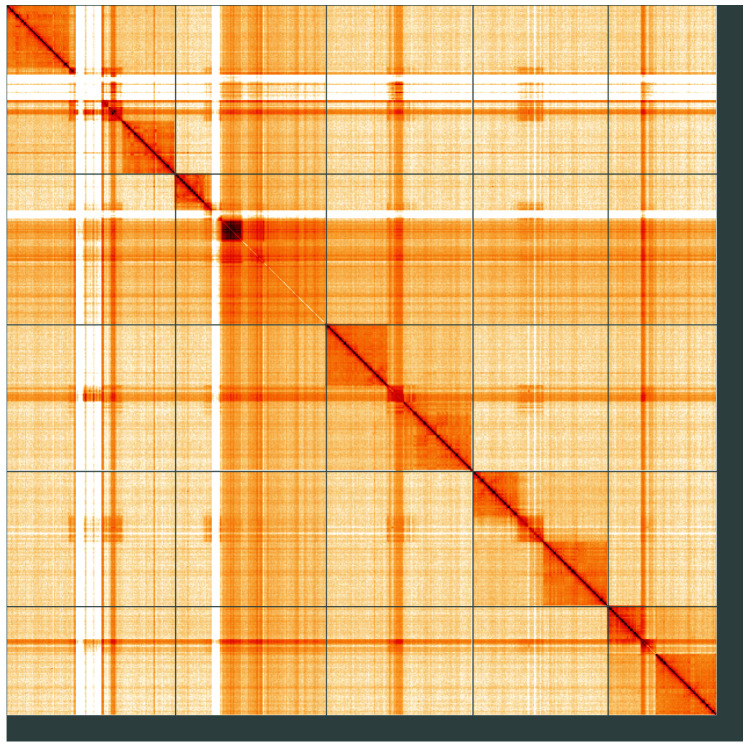
Genome assembly of *Pemphredon lugubris*, iyPemLugu1.1: Hi-C contact map of the iyPemLugu1.1 assembly, visualised using HiGlass. Chromosomes are shown in order of size from left to right and top to bottom. An interactive version of this figure may be viewed at https://genome-note-higlass.tol.sanger.ac.uk/l/?d=bxzUvAXrQlGeHrz_iSh0VQ.

The final assembly has a total length of 328.1 Mb in 1358 sequence scaffolds with a scaffold N50 of 64.8 Mb ( Table 1). The snailplot in Figure 2 provides a summary of the assembly statistics, while the distribution of assembly scaffolds on GC proportion and coverage is shown in Figure 3. The cumulative assembly plot in Figure 4 shows curves for subsets of scaffolds assigned to different phyla. Most (95.65%) of the assembly sequence was assigned to 5 chromosomal-level scaffolds, representing 5 autosomes. Chromosome-scale scaffolds confirmed by the Hi-C data are named in order of size ( Figure 5; Table 2). The assembly is haploid. The order and orientation of scaffolds are uncertain on chromosome 1 in the region 31.55 to 42.17 Mb, and chromosome 2 in the region 17.43 to 65.55 Mb. The mitochondrial genome was also assembled and can be found as a contig within the multifasta file of the genome submission.

**Table 1.** Genome data for *Pemphredon lugubris*, iyPemLugu1.1.

Project accession data		
Assembly identifier	iyPemLugu1.1	
Species	*Pemphredon lugubris*	
Specimen	iyPemLugu1	
NCBI taxonomy ID	2495172	
BioProject	PRJEB50731	
BioSample ID	SAMEA8603139	
Isolate information	iyPemLugu1, male: thorax (DNA sequencing), head (Hi-C scaffolding), abdomen (RNA sequencing)	
Assembly metrics *		*Benchmark*
Consensus quality (QV)	43.4	*≥ 50*
*k*-mer completeness	99.8%	*≥ 95%*
BUSCO **	C:96.8%[S:96.5%,D:0.4%],F:0.6%,M:2.6%,n:5,991	*C ≥ 95%*
Percentage of assembly mapped to chromosomes	95.65%	*≥ 95%*
Sex chromosomes	-	*localised homologous pairs*
Organelles	Mitochondrial genome: 15.88 kb	*complete single alleles*
Raw data accessions		
PacificBiosciences SEQUEL II	ERR8575361	
10X Genomics Illumina	ERR8571637, ERR8571634, ERR8571635, ERR8571636	
Hi-C Illumina	ERR8571639	
PolyA RNA-Seq Illumina	ERR8571638	
Genome assembly		
Assembly accession	GCA_933228935.1	
Span (Mb)	328.1	
Number of contigs	1786	
Contig N50 length (Mb)	21.6	
Number of scaffolds	1358	
Scaffold N50 length (Mb)	64.8	
Longest scaffold (Mb)	74.67	
Genome annotation		
Number of protein-coding genes	10,335	
Number of non-coding genes	2,411	
Number of gene transcripts	20,719	

* Assembly metric benchmarks are adapted from column VGP-2020 of “Table 1: Proposed standards and metrics for defining genome assembly quality” from ( Rhie *et al.*, 2021). ** BUSCO scores based on the hymenoptera_odb10 BUSCO set using version 5.3.2. C = complete [S = single copy, D = duplicated], F = fragmented, M = missing, n = number of orthologues in comparison. A full set of BUSCO scores is available at https://blobtoolkit.genomehubs.org/view/CAKOGF01/dataset/CAKOGF01/busco.

**Table 2.** Chromosomal pseudomolecules in the genome assembly of *Pemphredon lugubris*, iyPemLugu1.

INSDC accession	Chromosome	Length (Mb)	GC%
OW121815.1	1	74.67	44.0
OW121816.1	2	66.56	40.0
OW121817.1	3	64.77	43.5
OW121818.1	4	59.7	43.0
OW121819.1	5	48.19	42.0
OW121820.1	MT	0.02	17.5

The estimated Quality Value (QV) of the final assembly is 43.4 with *k*-mer completeness of 99.8%, and the assembly has a BUSCO v5.3.2 completeness of 96.8% (single = 96.5%, duplicated = 0.4%), using the hymenoptera_odb10 reference set ( *n* = 5,991).

Metadata for specimens, barcode results, spectra estimates, sequencing runs, contaminants and pre-curation assembly statistics are given at https://links.tol.sanger.ac.uk/species/2495172.

## Genome annotation report

The *Pemphredon lugubris* genome assembly (GCA_933228935.1) was annotated using the Ensembl rapid annotation pipeline ( Table 1; https://rapid.ensembl.org/Pemphredon_lugubris_GCA_933228935.1/Info/Index). The resulting annotation includes 20,719 transcribed mRNAs from 10,335 protein-coding and 2,411 non-coding genes.

## Methods

### Sample acquisition and nucleic acid extraction

A male *Pemphredon lugubris* (specimen ID Ox000901, ToLID iyPemLugu1) was netted in Wytham Woods, Oxfordshire (biological vice-county Berkshire), UK (latitude 51.77, longitude –1.31) on 2020-08-03. The specimen was collected and identified by Steven Falk (independent researcher) and preserved on dry ice.

The workflow for high molecular weight (HMW) DNA extraction at the WSI includes a sequence of core procedures: sample preparation; sample homogenisation, DNA extraction, fragmentation, and clean-up. In sample preparation, the iyPemLugu1 sample was weighed and dissected on dry ice ( Jay *et al.*, 2023). Thorax tissue was homogenised using a PowerMasher II tissue disruptor ( Denton *et al.*, 2023a). HMW DNA was extracted using the Automated MagAttract v1 protocol ( Sheerin *et al.*, 2023). HMW DNA was sheared into an average fragment size of 12–20 kb in a Megaruptor 3 system with speed setting 30 ( Todorovic *et al.*, 2023). Sheared DNA was purified by solid-phase reversible immobilisation ( Strickland *et al.*, 2023): in brief, the method employs a 1.8X ratio of AMPure PB beads to sample to eliminate shorter fragments and concentrate the DNA. The concentration of the sheared and purified DNA was assessed using a Nanodrop spectrophotometer and Qubit Fluorometer and Qubit dsDNA High Sensitivity Assay kit. Fragment size distribution was evaluated by running the sample on the FemtoPulse system.

RNA was extracted from abdomen tissue of iyPemLugu1 in the Tree of Life Laboratory at the WSI using the RNA Extraction: Automated MagMax™ *mir*Vana protocol ( do Amaral *et al.*, 2023). The RNA concentration was assessed using a Nanodrop spectrophotometer and a Qubit Fluorometer using the Qubit RNA Broad-Range Assay kit. Analysis of the integrity of the RNA was done using the Agilent RNA 6000 Pico Kit and Eukaryotic Total RNA assay.

Protocols developed by the Wellcome Sanger Institute (WSI) Tree of Life core laboratory have been deposited on protocols.io ( Denton *et al.*, 2023b).

### Sequencing

Pacific Biosciences HiFi circular consensus and 10X Genomics read cloud DNA sequencing libraries were constructed according to the manufacturers’ instructions. Poly(A) RNA-Seq libraries were constructed using the NEB Ultra II RNA Library Prep kit. DNA and RNA sequencing was performed by the Scientific Operations core at the WSI on Pacific Biosciences SEQUEL II (HiFi), Illumina HiSeq 4000 (RNA-Seq) and Illumina NovaSeq 6000 (10X) instruments. Hi-C data were also generated from head tissue of iyPemLugu1 using the Arima2 kit and sequenced on the Illumina NovaSeq 6000 instrument.

### Genome assembly, curation and evaluation

Assembly was carried out with Hifiasm ( Cheng *et al.*, 2021). One round of polishing was performed by aligning 10X Genomics read data to the assembly with Long Ranger ALIGN, calling variants with FreeBayes ( Garrison & Marth, 2012). The assembly was then scaffolded with Hi-C data ( Rao *et al.*, 2014) using YaHS ( Zhou *et al.*, 2023). The assembly was checked for contamination and corrected using the gEVAL system ( Chow *et al.*, 2016) as described previously ( Howe *et al.*, 2021). Manual curation was performed using gEVAL, HiGlass ( Kerpedjiev *et al.*, 2018) and Pretext ( Harry, 2022). The mitochondrial genome was assembled using MitoHiFi ( Uliano-Silva *et al.*, 2023), which runs MitoFinder ( Allio *et al.*, 2020) or MITOS ( Bernt *et al.*, 2013) and uses these annotations to select the final mitochondrial contig and to ensure the general quality of the sequence.

A Hi-C map for the final assembly was produced using bwa-mem2 ( Vasimuddin *et al.*, 2019) in the Cooler file format ( Abdennur & Mirny, 2020). To assess the assembly metrics, the *k*-mer completeness and QV consensus quality values were calculated in Merqury ( Rhie *et al.*, 2020). This work was done using Nextflow ( Di Tommaso *et al.*, 2017) DSL2 pipelines “sanger-tol/readmapping” ( Surana *et al.*, 2023a) and “sanger-tol/genomenote” ( Surana *et al.*, 2023b). The genome was analysed within the BlobToolKit environment ( Challis *et al.*, 2020) and BUSCO scores ( Manni *et al.*, 2021; Simão *et al.*, 2015) were calculated.

Table 3 contains a list of relevant software tool versions and sources.

**Table 3.** Software tools: versions and sources.

Software tool	Version	Source
BlobToolKit	4.1.7	https://github.com/blobtoolkit/blobtoolkit
BUSCO	5.3.2	https://gitlab.com/ezlab/busco
FreeBayes	1.3.1-17-gaa2ace8	https://github.com/freebayes/freebayes
gEVAL	N/A	https://geval.org.uk/
Hifiasm	0.15.3	https://github.com/chhylp123/hifiasm
HiGlass	1.11.6	https://github.com/higlass/higlass
Long Ranger ALIGN	2.2.2	https://support.10xgenomics.com/genome-exome/ software/pipelines/latest/advanced/other-pipelines
Merqury	MerquryFK	https://github.com/thegenemyers/MERQURY.FK
MitoHiFi	2	https://github.com/marcelauliano/MitoHiFi
PretextView	0.2	https://github.com/wtsi-hpag/PretextView
sanger-tol/genomenote	v1.0	https://github.com/sanger-tol/genomenote
sanger-tol/readmapping	1.1.0	https://github.com/sanger-tol/readmapping/tree/1.1.0
YaHS	1	https://github.com/c-zhou/yahs

### Genome annotation

The Ensembl gene annotation system ( Aken *et al.*, 2016) was used to generate annotation for the *Pemphredon lugubris* assembly (GCA_933228935.1). Annotation was created primarily through alignment of transcriptomic data to the genome, with gap filling via protein-to-genome alignments of a select set of proteins from UniProt ( UniProt Consortium, 2019).

### Wellcome Sanger Institute – Legal and Governance

The materials that have contributed to this genome note have been supplied by a Darwin Tree of Life Partner. The submission of materials by a Darwin Tree of Life Partner is subject to the **‘Darwin Tree of Life Project Sampling Code of Practice’**, which can be found in full on the Darwin Tree of Life website here. By agreeing with and signing up to the Sampling Code of Practice, the Darwin Tree of Life Partner agrees they will meet the legal and ethical requirements and standards set out within this document in respect of all samples acquired for, and supplied to, the Darwin Tree of Life Project.

Further, the Wellcome Sanger Institute employs a process whereby due diligence is carried out proportionate to the nature of the materials themselves, and the circumstances under which they have been/are to be collected and provided for use. The purpose of this is to address and mitigate any potential legal and/or ethical implications of receipt and use of the materials as part of the research project, and to ensure that in doing so we align with best practice wherever possible. The overarching areas of consideration are:

•   Ethical review of provenance and sourcing of the material

•   Legality of collection, transfer and use (national and international) 

Each transfer of samples is further undertaken according to a Research Collaboration Agreement or Material Transfer Agreement entered into by the Darwin Tree of Life Partner, Genome Research Limited (operating as the Wellcome Sanger Institute), and in some circumstances other Darwin Tree of Life collaborators.

## Data Availability

European Nucleotide Archive: *Pemphredon lugubris* (mournful wasp). Accession number PRJEB50731; https://identifiers.org/ena.embl/PRJEB50731 ( Wellcome Sanger Institute, 2022). The genome sequence is released openly for reuse. The *Pemphredon lugubris* genome sequencing initiative is part of the Darwin Tree of Life (DToL) project. All raw sequence data and the assembly have been deposited in INSDC databases. Raw data and assembly accession identifiers are reported in Table 1.

## Author information

Members of the University of Oxford and Wytham Woods Genome Acquisition Lab are listed here: https://doi.org/10.5281/zenodo.7125292.

Members of the Darwin Tree of Life Barcoding collective are listed here: https://doi.org/10.5281/zenodo.4893703.

Members of the Wellcome Sanger Institute Tree of Life Management, Samples and Laboratory team are listed here: https://doi.org/10.5281/zenodo.10066175.

Members of Wellcome Sanger Institute Scientific Operations: Sequencing Operations are listed here: https://doi.org/10.5281/zenodo.10043364.

Members of the Wellcome Sanger Institute Tree of Life Core Informatics team are listed here: https://doi.org/10.5281/zenodo.10066637.

Members of the Tree of Life Core Informatics collective are listed here: https://doi.org/10.5281/zenodo.5013541.

Members of the Darwin Tree of Life Consortium are listed here: https://doi.org/10.5281/zenodo.4783558.
