# Supplementary material for: The genome sequence of the Mournful Wasp, Pemphredon lugubris (Fabricius, 1793)
Source: Wellcome Open Res. 2024 Feb 23;9:93. [Version 1] doi: 10.12688/wellcomeopenres.20948.1 (PMC11364974; doi:10.12688/wellcomeopenres.20948.1)
Supplement: Peer review history

# Reviewer report: Reviewer response for version 1

**DOI:** 10.21956/wellcomeopenres.23175.r92038

**Published:** 2024-08-30

**Authors:** Josephine Paris

**Affiliations:** 1 Department of Health, University of L'Aquila, Coppito, Italy

This Data Note describes the assembly of the Mournful Wasp genome using PacBio HiFi and 10X Genomics Data. The Background contains sufficient information on the species' biology, including its distribution and its ecology. The genome presented is of good quality given the technology used and the annotation has been conducted by Ensembl. The data are available on the ENA under the specified project accession. Metadata and other assembly statistics are also available on TOL. I only have a few minor comments:

 1. Please provide a quantitative summary of how much of the assembly was scaffolded in the Abstract (i.e. 95.65%)

 2. Please clarify whether MITOS or MitoFinder was used for the mirogenome

Are sufficient details of methods and materials provided to allow replication by others?

Partly

Is the rationale for creating the dataset(s) clearly described?

Yes

Are the datasets clearly presented in a useable and accessible format?

Yes

Are the protocols appropriate and is the work technically sound?

Yes

Reviewer Expertise:

Non-model population genomics

I confirm that I have read this submission and believe that I have an appropriate level of expertise to confirm that it is of an acceptable scientific standard.

# Reviewer report: Reviewer response for version 1

**DOI:** 10.21956/wellcomeopenres.23175.r90614

**Published:** 2024-07-31

**Authors:** Juan Wulff

**Affiliations:** 1 NCSU, Raleigh, USA

**Major concerns**

 The work in general is very good and the method used for sequencing quite robust. The manuscript could be a bit more descriptive about some aspects, but I understand that it is more a report than a research paper. The main weakness of the study is that the annotation was completed using the Uniprot database without transcriptomes supporting the gene annotation. I believe that the quality of the genome assembly is very good but in terms of gene annotation should be considered a draft genome to be improved in the future.

**Minors**

- Title: I recommend adding the insect’ order and family to the title.
- “P. morio”: genera cannot be abbreviated if the complete species name was not mentioned before.

Are sufficient details of methods and materials provided to allow replication by others?

Yes

Is the rationale for creating the dataset(s) clearly described?

Yes

Are the datasets clearly presented in a useable and accessible format?

Yes

Are the protocols appropriate and is the work technically sound?

Yes

Reviewer Expertise:

Insect molecular biology

I confirm that I have read this submission and believe that I have an appropriate level of expertise to confirm that it is of an acceptable scientific standard.
